# 

**Published:** 1884-01

**Authors:** 


Vol. XXXVIII. JANUARY, 1884. No. 1.

441-

### THE DENTAL REGISTER

A MONTHLY JOURNAL,

LIBRARY INDEXED S.G.O.

DEVOTED TO THE INTERESTS OF THE PROFESSION.

EDITED BY

J. TAFT, D.D.S.

PUBLISHED BY

SPENCER & CROCKER,

OHIO DENTAL AND SURGICAL DEPOT,

Nos. 117, 119, 121 WEST FIFTH STREET, CINCINNATI, OHIO.

**TERMS:—$2.00 per Annum, in Advance. Single Copies, 25 cts.**

